# Challenges and Opportunities in Recruiting a Very Large Sample of Pregnant Individuals: Secondary Analysis of an Online Nationwide Randomized Controlled Trial

**DOI:** 10.2196/72580

**Published:** 2025-10-24

**Authors:** Breanne Laird, Sara Moyer, Jennifer Huberty, Susan Bodnar-Deren, Patricia Kinser

**Affiliations:** 1 College of Health Solutions, Arizona State University, 500 North 3rd Street, Tempe, AZ, 85004, United States, 1 9095574002; 2 Family and Community Health Nursing, School of Nursing, Virginia Commonwealth University, Richmond, VA, United States; 3 Fit Minded, Inc, Phoenix, AZ, United States; 4 Department of Sociology and Institute for Women's Health, College of Humanities and Sciences, Virginia Commonweath University, Richmond, VA, United States

**Keywords:** recruitment, digital health, perinatal health, mental health, maternal health

## Abstract

**Background:**

It is challenging to recruit vulnerable populations such as pregnant individuals, particularly during the perinatal period, which involves significant life changes and stressful situations that may create barriers to participation. Barriers to participation are even more prominent in historically marginalized populations, such as minoritized and low-income populations. Current literature is limited on recruitment methods, and specific activities may be best to recruit diverse pregnant individuals into online studies for the promotion of perinatal mental health.

**Objective:**

The aim of this paper is to describe recruitment methods and strategies used to recruit a large sample (n=1953) of diverse pregnant individuals to an online nationwide large-scale randomized controlled trial, the Mamma Mia Study.

**Methods:**

The Mamma Mia study is a multisite randomized controlled trial of an online- and mobile-based intervention for preventing and reducing perinatal depressive symptoms, based in the United States. The study intended to ensure a diverse national representation in the study population, with internal team demographic goals of at least 50% of participants identifying as a race or ethnicity other than White and at least 25% low-income (defined as a household income of less than US $50,000). Institutional review board–approved active and passive recruitment methods, both online nationally and in-person locally, were used to recruit eligible pregnant individuals.

**Results:**

Combining local and in-person with national and online recruitment methods allowed for successful recruitment of a large and diverse sample of pregnant individuals, despite the necessity for several pivots due to national events (eg, the COVID-19 pandemic). In addition, this layered approach allowed the study to continue during an unplanned world event and be responsive to pivoting to meet recruitment goals. Recruitment approaches and methods that were the most successful were establishing community partnerships both online nationally and in-person locally, dedicated research time to focus on recruiting historically marginalized groups for a more representative sample, allocation of study time and resources to recruitment preparation, and dedicated internal research team recruitment planning and tracking.

**Conclusions:**

Researchers should continue to publish and disseminate specific details about recruitment efforts and results, highlighting both aspects of success and lessons learned, as well as the pivot points in their recruitment methods for shared learning.

## Introduction

Studies that promote mental health and well-being in pregnant populations are often statistically underpowered due to small sample sizes that fail to meet recruitment goals [1-3]. It is challenging to recruit vulnerable populations such as pregnant women, particularly during the perinatal period, which involves significant life changes and stressful situations that create barriers to participation [1]. Barriers to participation are even more prominent in historically marginalized populations, such as minoritized and low-income populations [4,5]. Low recruitment rates in these groups are commonly associated with reported issues such as access to research sites due to location, time commitment required, hesitancy due to lack of trust and knowledge in the research process due to misinformation, and concerns for privacy and confidentiality [1-4]. Other extenuating circumstances, such as time of year (eg, holidays) and world events (eg, COVID-19 pandemic), can intensify these challenges to an even greater extent. Participation of underrepresented groups in perinatal mental health research is necessary to understand and mitigate health disparities and to have findings that are generalizable to the population at large [4]. Research teams must be strategic about their study design and recruitment methodologies to ensure an adequate and representative sample of pregnant individuals is obtained.

Conducting online and mobile-based interventions that promote mental health and well-being for pregnant women during pregnancy and postpartum may be a way to decrease barriers to participation associated with in-person research [2]. The use of digitally delivered mental health programs reduces participation barriers related to travel costs, access, time, and the potential health risks of the public [2]. However, online and mobile-based studies with pregnant populations still struggle to be representative of historically marginalized groups [6,7]. A commonly used recruitment method, in general, is face-to-face in-person local recruitment that uses recruitment methods such as printed recruitment materials (eg, flyers, brochures, and postcards) that can be used both actively (eg, flyer handed to potential participant) and passively (eg, potential participant sees a posted flyer) [1-9]. Online recruitment is another recruitment method that has been increasing in popularity due to digital advancements. Online recruitment methods commonly used to recruit study participants, including pregnant individuals, are digital flyer postings through social media, email, and digital newsletters. These online methods promote greater reach and dissemination of the study participation opportunity [2]. Preliminary studies suggest online recruitment methods may help researchers successfully reach pregnant women [2], but there are still challenges to recruiting individuals who are traditionally underrepresented in research, such as historically marginalized populations by race, ethnicity, income, geolocation, and education [6,7].

It is unclear what general recruitment methods (ie, in-person, online, active, and passive) and associated specific recruitment methods for each strategy are best to recruit diverse groups of pregnant people into online studies for the promotion of perinatal mental health. It is timely and important for researchers to share with each other best practices for ensuring appropriate recruitment of diverse pregnant individuals. Transparency about challenges and opportunities is critical, whereby researchers can share their experiences and lessons learned to better equip future research with the strategies and tools needed to conduct studies with samples of participants that are representative of the target population effectively and credibly. Therefore, the purpose of this paper is to describe recruitment methods and strategies used to recruit a large sample of diverse pregnant individuals to an online nationwide large-scale randomized controlled trial (RCT). We explore methods and strategies used for recruitment and the successes and failures of these over the course of 4 years.

## Methods

### Study Design

The Mamma Mia study is a multisite randomized controlled trial of an online- and mobile-based intervention for preventing and reducing perinatal depressive symptoms that was conducted in the United States. The Mamma Mia study protocol and methods were published elsewhere [10]

Participant recruitment for the Mamma Mia Study occurred in the United States and used nationwide and local recruitment efforts. Local recruitment occurred at 2 geographical hubs: the central east coast region of the United States (East Central [EC] hub in Richmond, Virginia, led by study authors affiliated with Virginia Commonwealth University) and the southwestern region of the United States (Southwest hub, led by study authors affiliated with Arizona State University).

The study intended to ensure a diverse representation in the study population, with internal team demographic goals of at least 50% of participants who identified as Black or African American, Asian or Pacific Islander, Native American or Alaska Native, other or 2 or more races, or Latino or Hispanic and at least 25% low-income (as defined as a household income of less than US $50,000). Local active and passive recruitment was used to complement national recruitment efforts, given that online recruitment can have varying success in reaching individuals from historically marginalized populations [11,12]. We sought to recruit 1950 pregnant individuals who were: (1) less than 25 weeks gestation, (2) at least 18 years of age, (3) able to speak, read, and understand English as the intervention was available in English, and (4) had the ability to access an internet- or mobile-based program (via computer, tablet, or smartphone), and had access to a phone number and email address for study-related and safety protocol–related check-ins. All data collection occurred via digital platforms (eg, REDCap; Research Electronic Data Capture) via participant self-report survey, including the data about demographic questions (eg, race, ethnicity, income, and geolocation) and recruitment method (eg, “How did you hear about this study?”).

### Recruitment Plan

This study was designed to integrate nationwide online recruitment alongside local passive and active recruitment at both the EC Hub and Southwest Hub. Based on our team’s experience with previous studies [13-16] in combination with extant literature [17,18], we expected that online recruitment may not accurately represent the diverse pregnant populations in the United States. Therefore, the recruitment plan for this study was intentionally designed to incorporate passive and active recruitment at local hubs in a layered recruitment plan.

In this study, we conceptualized “community” and “partnerships” as layered and dynamic, extending beyond formal health care and research settings. Consistent with frameworks for authentic community engagement [19], we understood “community” to include formal organizations (eg, Women Infants and Children clinics, community health resource centers, Birth in Color) as well as informal networks (eg, community health workers, peer advocates) embedded within the lived realities of pregnant individuals, particularly those from historically marginalized groups. “Partnerships” were not limited to advisory or consultative relationships; rather, they reflected shared efforts to co-develop culturally relevant recruitment strategies, build trust, and foster mutual investment in the study’s goals. By adopting this layered and relational approach, we sought to create a more inclusive, trust-centered recruitment model, better positioned to reach diverse pregnant populations across geographic regions.

### Recruitment Preparation

Recruitment preparation included: (1) developing a recruitment plan and strategy, (2) building credibility and trust with an online presence, and (3) a recruitment tracking plan. Recruitment methods in our recruitment plan included where recruitment should take place (eg, online, in-person, and specific locations), who might have access to the target population (community partners), contact strategies for potential partners (email, phone, and social media), and developing recruitment materials (eg, flyers and recruitment scripts for both in-person and online communication). Community partner outreach and support included connecting with potential online community partners who would be willing to share about the study via their own established online platforms (eg, listserv emails and social media postings). Other recruitment preparation methods included developing a study website and developing social media accounts. These study-specific websites and accounts were used as landing pages to build credibility and trust with an online presence. Recruitment tracking was set up using the data collection tool REDCap, a secure web-based data collection and management system. Our recruitment tracking collected data related to the type of recruitment method each enrolled participant endorsed (eg, how did you hear about this study) in addition to tracking data about the type of recruitment material (eg, which flyer) was used throughout the recruitment period.

### Recruitment Methods

#### Internal Team Recruitment Strategies and Tracking

Internal team strategies for planning and tracking recruitment methods used included weekly team meetings for brainstorming, planning, and goal setting, as well as constant consideration of feedback from the community. Additional internal team methods included internal team accountability, such as weekly and monthly data updates to monitor progress and recruitment tracking (inclusive of our internal diversity benchmarks, how participants heard about the study, etc). Study team members also attended webinars and training focused on health equity and social justice to be able to design recruitment strategies and material most appropriate for collaborating with community partners and recruiting our target demographics.

#### Study Eligibility and Intake Procedures

An essential aspect of data integrity for studies that use online recruitment methods is data integrity and sample validity by ensuring that enrolled study participants are a potential human research participant (eg, not a bot) and they are accurately representing themselves during the enrollment screening process [8,20]. Therefore, we implemented the following layered strategies. First, we posted the link to our study-specific website (rather than the direct survey link) in public open forum online spaces. Second, we activated a reCAPTCHA (Completely Automated Public Turing test to tell Computers and Humans Apart) tool on the eligibility screening form on REDCap. Third, we required an enrollment phone call during which the research team reviewed the consent form and had participants verify several free-text items from the enrollment form, including where they heard about the study. This allowed us to track our recruitment efforts, including snowball recruitment. Fourth, we cross-referenced each new enrollment participant’s email address with our recent enrolled database to screen for multiple concurrent enrollments by the same individual.

### Ethical Considerations

This study, including all recruitment materials and study plans, was approved by the Virginia Commonwealth University Institutional Review Board (IRB; HM20017197), with reliance on the VCU IRB by the Arizona State University IRB. All participants provided electronic consent prior to participating in the study. To protect privacy and confidentiality, all data collected were deidentified, with personal information securely stored and accessible only to authorized personnel. Financial compensation was offered to all study participants (up to US $140 in gift cards) and provided for each study task (eg, survey completion).

## Results

### Overview

Our final sample consisted of n=1953 participants. The study sample included participants who self-identified as African American or Black (367/1953, 18.8%), Asian or Pacific Islander (95/1953, 4.9%), Native American or Alaska Native (14/1953, 0.7%), White (1255/1953, 64.3%), Other (61/1953, 3.1%), and Latino or Hispanic (158/1953, 8.1%). In addition, this sample consisted of at least one participant from each of the 50 states and the District of Columbia, and participants were distributed across the nation with regional representation as follows: 17% from the Northeast, 20.6% from the Midwest, 38.9% from the South, and 23.6% from the West. The majority of participants were recruited from Virginia (611/1953, 31.3%), California (132/1953, 6.8%), North Carolina (86/1953, 4.4%), Texas (74/1953, 3.8%), and New York (71/1953, 3.6%). Our sample is comparable to the US Census population data of women of reproductive age (15-44 years) as of July 2023 (13.4% Black or African American, 6.8% Asian or Pacific Islander, 1.2% Native American or Alaska Native, 51.8% White, 13.7% two or more races, and 22.2% Latino or Hispanic) [21].

As mentioned previously, the team identified internal benchmarks for race, ethnicity, and income to track as a metric of study sample diversity. In addition, the team tracked how many participants heard about the study from each recruitment method (see Table 1): online and national recruitment (including social media posts and email blasts from community partner organizations), in-person local recruitment (including active and passive recruitment methods surrounding each regional hub), and snowball recruitment (including participants that actively spread the word to their connections regarding the study).

**Figure 1.**
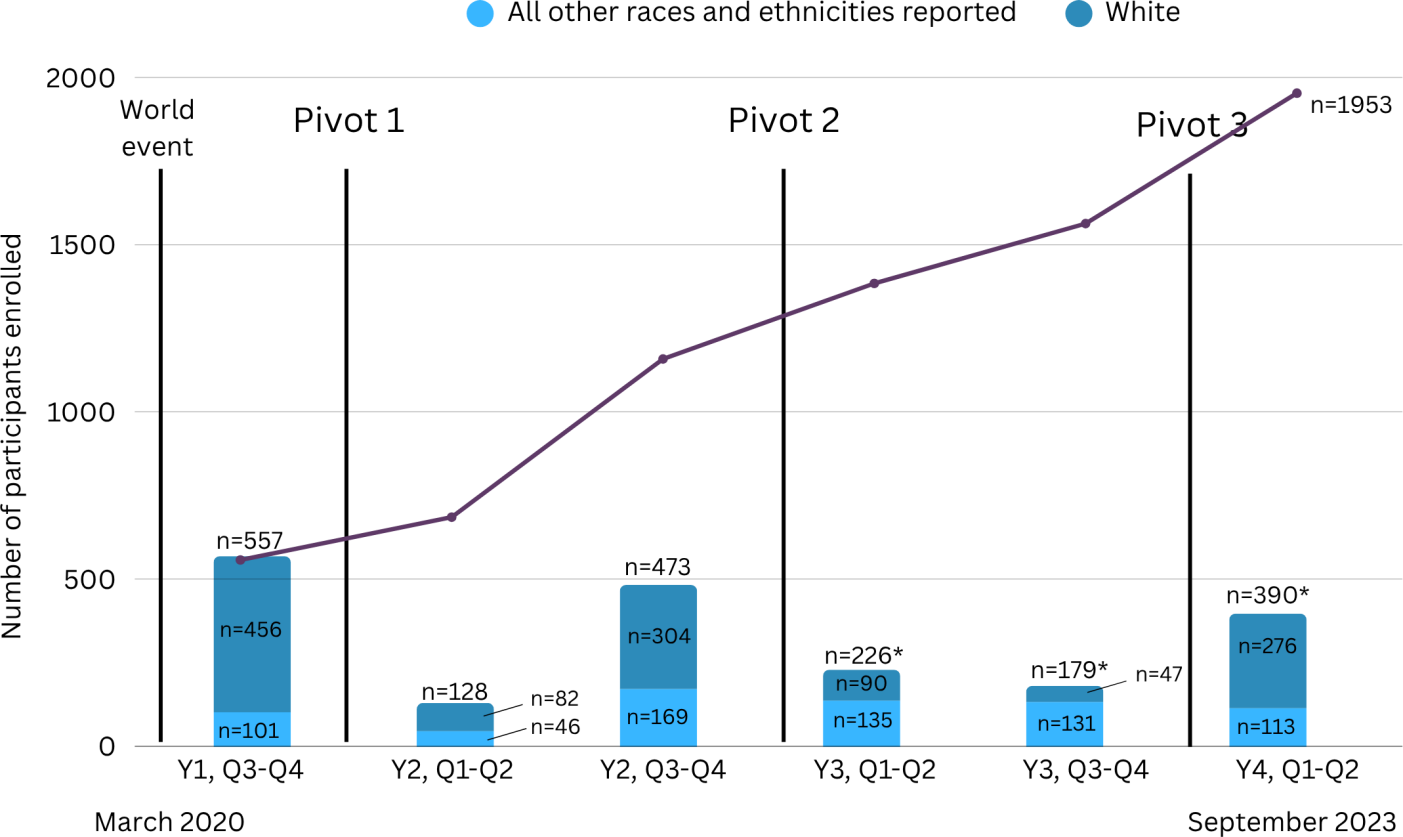
Overall recruitment data. *: n=1 with missing race and ethnicity data.

Our team began recruitment on October 15, 2020 (Y 1 Q3) and ended on September 1, 2023 (Y 4 Q2). Figure 1 depicts overall recruitment data for this RCT, along with world events and pivots during recruitment, and benchmark details by year/quarter.

**Table 1.** Recruitment timeline.

Grant year	Year 1		Year 2		Year 3		Year 4	Total
Quarters	1-2	3-4	1-2	3-4	1-2	3-4	1-2	—
Recruitment goals, n (%)	—^a^	162 (8.3)	366 (18.8)	366 (18.8)	366 (18.8)	366 (18.8)	324 (16.6)	1950 (100)
Recruitment actual, n (%)	—	557 (28.5)	128 (6.6)	473 (24.3)	226 (11.6)	179 (9.2)	390 (20)	1953 (100.2)
Internal team benchmark tracking								
Race/ethnicity, n (%)								
Any race or ethnicity reported other than White	—	101 (18.1)	46 (35.9)	169 (35.7)	135 (59.7)	131 (73.2)	113 (29)	695 (35.6)
White	—	456 (81.9)	82 (64.1)	304 (64.3)	90 (39.8)	47 (26.3)	276 (70.8)	1255 (64.3)
Missing	—	—	—	—	1 (0.4)	1 (0.6)	1 (0.31)	3 (0.2)
Income, n (%)								
<US $50,000/year	—	78 (14)	22 (17.2)	105 (22.2)	95 (42)	97 (54.2)	73 (18.7)	470 (24.1)
>US $50,000/year	—	469 (84.2)	104 (81.3)	364 (77.0)	126 (55.8)	80 (44.7)	312 (80)	1455 (74.5)
Missing	—	10 (1.8)	2 (1.5)	4 (0.8)	5 (2.2)	2 (1.1)	5 (1.3)	28 (1.4)
Recruitment strategies, n (%)								
National	—	403 (72.4)	74 (57.8)	35 (7.4)	70 (31)	29 (16.2)	239 (61.3)	850 (43.5)
Southwest hub	—	1 (0.2)	0 (0)	3 (0.6)	13 (5.8)	8 (4.5)	1 (0.3)	26 (1.3)
EC hub^b^	—	83 (14.9)	41 (32)	158 (33.4)	132 (58.4)	124 (69.3)	117 (30)	655 (33.5)
Snowball	—	70 (12.6)	13 (10.2)	277 (58.6)	11 (4.9)	18 (10.1)	33 (8.5)	422 (21.6)

a Not applicable.

b EC hub: East Coast region local hub.

### World Event

Of note, this study launched in the Spring of 2020, which co-occurred with the major world event of the SARS-CoV-2 (COVID-19) pandemic; this is noted as “World Event” in Figure 1. As such, no direct in-person recruitment was possible in local clinics and community events. However, because our recruitment plan included a dual approach of online and in-person recruitment, the study was able to continue operating when many research studies had to completely suspend operation.

### Pivots in Recruitment Approaches

Pivot 1: After deploying online recruitment methods (eg, social media) that yielded high recruitment and enrollment numbers initially, we noticed a majority of these participants identified as White and reported higher income brackets. The team had originally planned to integrate in-person recruitment at each of our local hubs to account for this potential issue; however, due to pandemic-related restrictions, that was not possible. To be able to reach our diversity goals, as a team, we enacted Pivot 1. In this pivot, we aimed to focus our online recruitment efforts on potential participants who identified as a person of color by (1) connecting with online community partners on social media who specifically focused their content on pregnant individuals identifying as people of color, (2) updating our recruitment materials and images, and (3) adding language adjustments to our recruitment materials and study website specifically highlighting the study’s goal of including pregnant individuals identifying as people of color.

Pivot 2: As pandemic-related restrictions shifted, our team enacted Pivot 2. In this pivot, we allocated intentional time and efforts toward in-person recruitment at both local hubs in addition to continued online and national efforts. Continuing the focus to recruit a diverse and representative sample, we intentionally focused on posting recruitment materials and attending in-person events in a variety of community-based locations. For example, our study teams attended community events such as powwows for local indigenous tribes in rural areas of Virginia and multicultural festivals in several urban areas in central Virginia. We also collaborated with our local community partners who held community baby showers and other celebratory events for the pregnant and postpartum community in Virginia (eg, Black Maternal Mental Health Week). We activated our professional and clinical network to engage in active recruitment through presence at our academic clinical partner sites. Specific to our academic clinical partner site, we obtained a waiver of consent through the IRB for prescreening of medical records. In this prescreening process, no data was recorded about potential participants. This process allowed team members to actively engage with potential participants who met the team’s internal benchmarks for race, ethnicity, and income.

Also, during this pivot, we mentioned that participants were welcome to share about the study with their communities or other pregnant individuals during the enrollment phone call. This was an organic process that arose after several participants asked permission to share with friends or family, or even via private social media groups (eg, Reddit). At the time of Pivot 2, we intentionally integrated this discussion into the enrollment call script with every newly enrolled participant with a simple offering of, “Also, you are welcome to share about this study with anyone else who may want to hear about it. You can share our website or are welcome to give them my (the research coordinator’s) contact info if they would like additional information.” Having an easily shareable study-specific website that served as a direct way to share the study was critical to this process.

Pivot 3: Throughout the study, our team tracked enrollment by state, given an internal team goal to have representation across the nation. As our team neared our goal completion date for recruitment, we completed a final overall review of our study enrollment progress to date and noticed that we had minimal representation from the Midwest region of the United States. As such, we engaged with an online community partner who had tailored social media content for pregnant women in the Midwest to share study recruitment materials. In addition, to meet total enrollment goals, we re-engaged with study partners about re-sharing recruitment materials.

## Discussion

### Overview

The purpose of this paper was to describe recruitment methods and strategies used to recruit a large sample (n=1953) of diverse pregnant individuals to an online nationwide large-scale RCT. The intent of sharing these details is to inform other research studies intending to conduct similar large-scale online studies. The study was successful in meeting total enrollment goals and partially successful in meeting goals of enrolling participants identifying as Black or African American, Asian or Pacific Islander, Native American or Alaska Native, other or two or more races, or Latino or Hispanic, and low-income. We discuss our experiences and future considerations for researchers below.

A combination of in-person and local, online and national, and passive and active recruitment, combined with internal team recruitment planning and tracking strategies, helped us reach our recruitment goals in a timely manner. The most successful recruitment methods were: (1) establishing community partnerships both in-person locally and online nationwide, (2) ensuring team-wide dedication to recruiting individuals from historically marginalized groups, and (3) maintaining a nimble internal team approach. Recruitment approaches that did not yield many participants included mailed postcards, newsletters, and “boosted” posts on Facebook (Meta Inc) and Instagram (Meta Inc). In line with extant literature, mailed postcards and newsletters often result in low response rates (typically <10%) and overrepresent older, White, and more educated populations compared to more active approaches [22]. Similarly, “boosted” social media posts may yield high traffic but produce lower eligibility and completion rates [23,24].

We established community partnerships by engaging with physicians and perinatal-focused organizations in person and by working with individuals and organizations online nationally who had large followings and were willing to share information about our study. Given our experiences and the extant literature, we believe that research teams should strive for sustained, bi-directional engagement when building relationships with community partners both in person and online; intentionally focusing on relationships based on aligned missions that can grow toward true engagement and collaboration through a program of research rather than a one-time recruitment engagement. We ensured team-wide dedication to recruitment goals through internal team recruitment strategies, including (1) intentional dedication to recruiting diverse participants, (2) recruitment preparation through website and social media development, (3) online and social media community partner feedback collaborations, and (4) remaining flexible and open to pivoting recruitment efforts and goals based on recruitment performance and community feedback.

### In-Person and Local Recruitment

#### In-Person and Local Methods

In-person and local recruitment methods included building collaborations with community partners such as practitioners (eg, clinic partnerships), researchers (eg, other research teams working with perinatal populations), businesses or individuals (eg, local and regional organizations or members focused on perinatal health), events, and mailed postcards. For community partners who were a part of the study team, financial compensation was provided for their time and efforts. Although funding was not available for every community partner, some other ideas for extending gratitude to community partners who were interested included adding their logo on the study website with a clickable link; the research team collaborating to create educational content to be shared through the organization (eg, website, blog, and social media); and sharing the community partner’s content or social media posts as mutually agreed upon and appropriate by both the study team and the community partner.

The use of purposeful in-person recruitment allows for focused recruitment of diverse populations in the community via community partnerships and professional networks. In-person methods that were most successful included the presence of research personnel in community and clinical settings commonly attended by pregnant individuals. Our research team established new relationships and expanded existing relationships with local primary care and obstetric clinics and organizations (eg, Women Infants and Children Program, Birth in Color RVA, Postpartum Support Virginia, Urban Baby Beginnings, Huddle Up Moms). These methods are consistent with previous literature suggesting that obstetrician and midwife support is essential for introducing patients to research studies for patients to feel comfortable participating in clinical research while pregnant [25]. When applicable, research team presence in clinic settings and taking the time to meet with clinic professionals to present study methods and answer questions gave our team the opportunity to be fully integrated in prenatal clinical settings.

Most importantly, this study included collaboration with a local community partner as a part of the research team, and this community partnership was an essential part of recruitment planning, recruitment material development, and community engagement planning at the local EC hub. For example, our primary community partner, “Birth in Color,” provides a myriad of services to pregnant women of color—from community baby showers, to parenting classes, to prenatal yoga, including events held in public housing communities. In addition, the study team sought additional community partner relationships with organizations serving pregnant women at the local level, developing relationships for bidirectional support, including supporting community events with volunteers. Research team members’ presence alongside community partners in community spaces is a first step toward community engagement and collaboration to develop research that is integrated into the community. In this way, research teams can include individuals who may not have been included in research through more typical clinical avenues; team members can answer questions in real-time and build relationships with potential participants [25].

### Online and National Recruitment

Online national recruitment methods included engaging with community partners online to build relationships, credibility, and trust with influencers and leaders of online communities that engage with diverse pregnant individuals in the United States. Online community partners included nonprofit organizations that shared our recruitment materials via their email listservs or websites and social media sharing by individuals or groups with large followings on various social media platforms. Additional recruitment strategies included paying for advertisements and “boosted” posts, as well as creating a study website for interested individuals to access information about the study and engage in enrollment activities. The study website included research team and study funding transparency, study coordinator contact information for questions, and information about community partners to support credibility and trust.

The greatest benefit of online recruitment methods we observed was being able to connect with community partners who treat, advocate for, or serve the target population nationwide. These methods allowed us to reach diverse populations and increase the generalizability of research findings. Online community partner outreach and support can begin during the project planning phase, grant development phase, and during the IRB approval stage. Preparation support includes letters of support or verbal (eg, email) agreement and receiving feedback on recruitment language and images and referrals to other potential community partners. Asking community partners to follow the study’s social media accounts can be a first step to begin establishing rapport in the community the research is targeting. Once community partners agree to collaborate, logos made clickable to partners’ websites or social media accounts can be uploaded to the study website. Securing online relationships with community partners is important for trust and credibility to be felt by the potential participant [21]. For example, multiple participants communicated they had signed up for our study because a trusted health care provider or public figure had shared the Mamma Mia study on their social media account. Our study website and social media presence allowed the community partners to direct potential participants to places online where they could learn more about the study, find contact information to ask questions, and see other community partners and followers who support the research. Researchers should consider following credible individuals in their field of study on their study social media accounts and ask community partners to follow the account back, effectively creating a well-integrated online community. Of note, some larger, national groups and organizations as well as nonprofits that serve both pregnant individuals and underserved groups were less likely to share via online platforms than smaller regional or local groups for a myriad of reasons; for example, some had multiple other concurrent social media campaign priorities or other research they were supporting, while others had a mission that did not align with the current recruitment goals, and some never responded or stopped responding to our outreach. Research teams may consider this when planning for engaging with online and social-media partners, as local and regional organizations with an online presence may be more impactful for study recruitment purposes.

The use of the internet for both recruitment of study participants and tracking recruitment efforts can be a cost-effective and high-yield strategy [2,8,20,26]. More specifically, the use of social media, email list-servs, websites, and online data collection tools can be highly effective online recruitment methods [8,20]. However, the technology literacy of team members and partners can impact the success of these strategies. Study teams who use online and digital recruitment strategies must be educated on social marketing and how a social media platform works (eg, how the algorithm works), how to best communicate with potential partners via email versus direct messages on social media, how to design and manage a website, and how to track recruitment efforts digitally for insight into what is working and what is not. Researchers should be aware of the level of experience and technology literacy of their teams and consider training team members in online strategies before study recruitment begins, even if they are experienced in study recruitment. Further, if the target population of a research study is historically marginalized populations, it is critical to ensure teams are trained in health equity and social justice concepts before starting recruitment activities. This training is essential for all health research professionals.

Partnering with individuals and organizations who already have established large followings can help spread the word about the study opportunity to a greater degree, effectively reaching the target potential participant population much more effectively than sharing via the research-specific accounts. In addition, when working with pregnant individuals, it is important to acknowledge that many participants wanted to share with others in their own communities about the research participation opportunity, leading to a snowball recruitment effect. Having relationships with community partners nationwide can increase recruitment reach, leading to more diverse samples that are more representative of the general population.

### Recruitment Materials

IRB-approved recruitment materials were used for both in-person and online recruitment, as well as for our active and passive recruitment methods. Recruitment materials included, but were not limited to, flyers, brochures, email scripts, images optimized for digital use, short videos, and social media post-scripts. Graphic design software was used to create study flyers, brochures, and digital graphics. All recruitment materials included inclusive images intended to reflect the many types of pregnant individuals. All recruitment materials, including emails without images, presented appropriate IRB-approved wording that had been cocreated with a consulting community partner. Recruitment materials were then updated and improved based on feedback from community partners, participants, and potential participants.

Recruitment material design and recruitment language are essential to successful study enrollment. The content of both paper and online recruitment materials must be professional, eye-catching, and well-received by the intended population [2]. Research teams should also be flexible when working with social media partners and consider methods to effectively and efficiently meet the needs of both the partners and the IRB to streamline this process. For example, simple techniques such as flexibility around the color scheme of the digital graphic used can be impactful, allowing the social media partner to keep their posts “on brand.” Researchers should seek feedback from their community partners during the design phase of recruitment materials.

Collaboration with community partners should occur regarding the recruitment flyers and digital graphics, email and social media wording used, as well as the design and information on a study’s website. For example, we collaborated with the community partner on our team to develop study recruitment materials and integrated their valuable feedback before launching study recruitment. This is particularly important when recruiting vulnerable and historically marginalized populations. Researchers must acknowledge historical exclusion and well-earned mistrust by medical and research communities and move toward language and images in recruitment materials that are intentionally inclusive [27]. Researchers should make sure that all study materials appropriately disclose the study purpose, personnel, and funding to support transparency and trust between the researchers and potential participants, and they should be open to constructive feedback from the community.

### Internal Team Support

Study recruitment cannot be successful without internal team strategies that emphasize training and accountability [28]. Researchers should consider frequent team meetings that include planning and goal-setting that align with the study timeline, recruitment data reporting based on goals, accountability for meeting goals, and being open and flexible to pivoting recruitment strategies as needed. Our internal team strategies are consistent with the literature that demonstrates recruiting historically marginalized pregnant women is most successful when studies combine in-person and remote recruitment with internal team coordination methods [17,18]. Recruitment management that includes preparation, training, and tracking will also ensure that various strategies are being used correctly and are providing the results intended. Insights from those who have recruited participants during pregnancy underscore that training in recruitment strategies can reduce gatekeeping and enable flexible adaptation [29].

### Recruitment Cost

Community partners often prefer and sometimes require financial support for their efforts. Although in-person recruitment has been found to be less cost-effective than online methods [2,30], we found that the typical cost for social media partners to advertise about the study through a website, social media post, or series ranged from US $150-US $7000. Studies planning to incorporate online advertising as part of their recruitment strategies should plan for this expenditure in the budget planning phase. Because this type of recruitment funding is often limited or not included in grant proposals at all, other forms of compensation should also be considered. For example, potential partners may find it valuable if direct links to their website or social media accounts are included on the study website; partners may also appreciate having their information shared with study participants through newsletters, blogs, and study landing pages. These strategies may allow researchers to reciprocate support with partners despite limited funding. We recommend that researchers plan ahead for and justify these costs for recruitment preparation time, active efforts, and costs in grant proposals.

### Protecting Data Integrity and Sample Validity

Ensuring sample validity, and therefore protecting data integrity, is of paramount concern for online studies using online recruitment strategies. Online studies should have systems in place to detect both robotic or spam involvement as well as deceptive enrollment (eg, human participants who do not meet the study enrollment criteria that may use deception in eligibility screening in order to participate in the study). In this study, useful strategies included sharing a study-specific website rather than the direct enrollment survey link via social media, implementing a reCAPTCHA tool on the eligibility screening survey, and requiring an enrollment and consent verification phone call before confirming enrollment, where participants were required to verify free-text items from the eligibility screener. Although not done in our study, we suggest that researchers may consider additional approaches such as (1) adding hidden fields to eligibility surveys that will be “seen” and filled out by robotic or spam respondents but not human participants, (2) activating a timestamp on online surveys to evaluate time spent completing each survey, and (3) requiring a video call for enrollment and consent verification [8,20]. Future studies should consider the tension between data integrity and ensuring that study enrollment procedures are not overly burdensome for participants.

Our findings confirm and extend previous work demonstrating the value of a multiapproach, community-engaged recruitment strategies that integrate both digital and in-person methods to reach underserved populations. While previous studies have suggested the efficacy of social media in perinatal research recruitment [31], our findings suggest the use of online methods alone, without community partners, may not be sufficient to meet diversity goals [31]. These findings align with concerns in recent literature of a digital divide and underrepresentation of marginalized populations in digital health research [20,32].

### Limitations and Future Considerations

While we were partially successful in recruiting a diverse sample, we did not fully achieve all of our internal benchmark goals. This study encountered several limitations to recruitment that are important to consider when interpreting the findings and planning for future research. First, using a low-income benchmark of US $50,000 may have been too high for some US regions, potentially limiting the generalizability of our findings for low-income individuals. Second, the study was conducted exclusively online and in English, which may have been a barrier to participation from non-English speaking populations or low-income individuals who may not have access to a tablet, smartphone, or computer. Future research should consider providing materials in multiple languages, offering tech support, or providing study devices to increase accessibility. Third, the recruitment period overlapped with the COVID-19 pandemic, which limited the ability to conduct in-person recruitment activities during the early phases of the study. Although the pivot to online recruitment was successful, the inability to rely on traditional in-person methods may have impacted the diversity and reach of the study sample. Fourth, our team encountered several barriers associated with online community partnerships. We recommend that research teams engage with online and social media partners early on, particularly in the grant budget planning phase, to allocate adequate budget line items for successful partnership compensation. Future research should consider the demographics of their local communities, resources for supporting local entities in assisting in recruitment, as well as the intended goals for the study and how dissemination of findings will directly impact the population being studied when deciding where and how to recruit.

Fifth, despite our efforts to recruit a geographically diverse sample, representation from some regions, such as the Midwest, was lower than anticipated. While we adapted our recruitment strategies to targeted community partnerships and online outreach, this imbalance highlights the ongoing need for tailored region-specific approaches to recruitment. Finally, we used multiple approaches to prevent fraudulent or duplicate enrollments, and this may add administrative burden that may not be feasible for all research teams. Additional research into scalable, automated approaches to ensure data integrity is needed.

### Conclusion

Recruiting participants to research studies is a complex and ever-changing process due to the importance of sample size and diversity, as well as advancements in technology. Best recruitment results for recruiting very large samples of historically marginalized groups to online and mobile-based RCTs are achieved with an integration of both in-person and online recruitment. This paper aimed to describe the recruitment plan, inclusive of recruitment methods, approaches, and strategies, used to recruit a large sample of diverse pregnant individuals to an online mobile-based RCT. Combining local and in-person with national and online recruitment methods allowed for successful recruitment of a large sample of pregnant individuals. In addition, this layered approach allowed the study to continue during an unplanned world event and be responsive to pivoting to meet recruitment goals. Recruitment approaches and methods that were the most successful were establishing community partnerships both online nationally and in-person locally, dedicated research time to focus on recruiting historically marginalized groups for a more representative sample, allocation of study time and resources to recruitment preparation, and a dedicated internal research team for recruitment planning and tracking. Researchers should continue to publish and disseminate their recruitment efforts and results, highlighting both aspects of success and lessons learned, as well as the pivot points in their recruitment methods for shared learning. As such, future studies may reach their intended sample sizes with appropriate, diverse representation of the population intended to benefit from the study findings.

## Abbreviations

CAPTCHA: Completely Automated Public Turing test to tell Computers and Humans Apart
EC: East Central
IRB: Institutional Review Board
RCT: randomized controlled trial
REDCap: Research Electronic Data Capture
VCU: Virginia Commonwealth University

## References

[1] Frew PM, Saint-Victor DS, Isaacs MB (2014). Recruitment and retention of pregnant women into clinical research trials: an overview of challenges, facilitators, and best practices. Clin Infect Dis.

[2] Cochrane KM, Hutcheon JA, Karakochuk CD (2022). Strategies for improving recruitment of pregnant women to clinical research: an evaluation of social media versus traditional offline methods. Digit Health.

[3] Hulsbosch LP, Nyklíček I, Potharst ES, Meems M, Boekhorst M, Pop VJM (2020). Online mindfulness-based intervention for women with pregnancy distress: design of a randomized controlled trial. BMC Pregnancy Childbirth.

[4] Committee on Improving the Representation of Women and Underrepresented Minorities in Clinical Trials and Research, Committee on Women in Science, Engineering, and Medicine, Policy and Global Affairs, National Academies of Sciences, Engineering, and Medicine (2022). Improving Representation in Clinical Trials and Research: Building Research Equity for Women and Underrepresented Groups. National Academies Press.

[5] Louis-Jacques AF, Heuberger AJ, Mestre CT (2023). Improving racial and ethnic equity in clinical trials enrolling pregnant and lactating individuals. J Clin Pharmacol.

[6] Turner BE, Steinberg JR, Weeks BT, Rodriguez F, Cullen MR (2022). Race/ethnicity reporting and representation in US clinical trials: a cohort study. Lancet Reg Health Am.

[7] Krukowski RA, Ross KM, Western MJ (2024). Digital health interventions for all? Examining inclusivity across all stages of the digital health intervention research process. Trials.

[8] Pozzar R, Hammer MJ, Underhill-Blazey M (2020). Threats of bots and other bad actors to data quality following research participant recruitment through social media: cross-sectional questionnaire. J Med Internet Res.

[9] Rider A, Aubry C, Moyer S, Kinser P (2019). Perspectives on successes and challenges in the recruitment and retention of pregnant women in a research study. Clin Res (Alex).

[10] Kinser P, Jallo N, Huberty J (2021). Study protocol for a multisite randomized controlled trial of an internet and mobile-based intervention for preventing and reducing perinatal depressive symptoms. Res Nurs Health.

[11] Lee EW, Denison FC, Hor K, Reynolds RM (2016). Web-based interventions for prevention and treatment of perinatal mood disorders: a systematic review. BMC Pregnancy Childbirth.

[12] Andersson G, Titov N, Dear BF, Rozental A, Carlbring P (2019). Internet-delivered psychological treatments: from innovation to implementation. World Psychiatry.

[13] Huberty J, Matthews J, Leiferman JA, Lee C (2018). Use of complementary approaches in pregnant women with a history of miscarriage. Complement Ther Med.

[14] Huberty J, Puzia M, Green J, Stecher C (2021). Mental health and meditation practices of pregnant women during COVID-19. Obstet Gynecol Res.

[15] Green J, James D, Larkey L (2021). A qualitative investigation of a prenatal yoga intervention to prevent excessive gestational weight gain: a thematic analysis of interviews. Complement Ther Clin Pract.

[16] Green J, Neher T, Puzia M, Laird B, Huberty J (2022). Pregnant women’s use of a consumer-based meditation mobile app: a descriptive study. Digit Health.

[17] Rokicki S, Gobburu A, Weidner M (2025). Barriers and strategies for recruitment of pregnant women in contemporary longitudinal birth cohort studies. BMC Med Res Methodol.

[18] Goldstein E, Bakhireva LN, Nervik K (2021). Recruitment and retention of pregnant women in prospective birth cohort studies: a scoping review and content analysis of the literature. Neurotoxicol Teratol.

[19] Blumenthal DS (2006). A community coalition board creates a set of values for community-based research. Prev Chronic Dis.

[20] Loebenberg G, Oldham M, Brown J (2023). Bot or not? Detecting and managing participant deception when conducting digital research remotely: case study of a randomized controlled trial. J Med Internet Res.

[21] (2023). NST-EST2023-POPCHG2020-2023: Annual estimates of resident population change for the United States, states, District of Columbia, and Puerto Rico and state rankings: April 1, 2020 to July 1, 2023. https://www2.census.gov/programs-surveys/popest/technical-documentation/file-layouts/2020-2023/NST-EST2023-POPCHG2020_2023.pdf.

[22] Ley C, Duan H, Parsonnet J (2023). Recruitment into antibody prevalence studies: a randomized trial of postcards vs. letters as invitations. BMC Med Res Methodol.

[23] Pekarsky C, Skiffington J, Leijser LM, Slater D, Metcalfe A (2022). Social media recruitment strategies to recruit pregnant women into a longitudinal observational cohort study: usability study. J Med Internet Res.

[24] Childers-Rockey ZA, Flesher EA, Stephens JI, Barton NK, Waldron ME, Rioux C (2025). Recruitment through social media ads and videocalls: cost, effectiveness, and lessons from the experiences of pregnancy study. Am J Epidemiol.

[25] Sutton EF, Cain LE, Vallo PM, Redman LM (2017). Strategies for successful recruitment of pregnant patients into clinical trials. Obstet Gynecol.

[26] Surdam J, Daly B, Fulton S (2020). Recruitment strategies for nurse enrollment in an online study. Nurs Res.

[27] Israel BA, Wallerstein N, Duran B, Oetzel JG, Minkler M (2017). Community-Based Participatory Research for Health.

[28] Cranfill JR, Freel SA, Deeter CE (2022). Development and evaluation of a novel training program to build study staff skills in equitable and inclusive engagement, recruitment, and retention of clinical research participants. J Clin Transl Sci.

[29] Hanrahan V, Gillies K, Biesty L (2020). Recruiters’ perspectives of recruiting women during pregnancy and childbirth to clinical trials: a qualitative evidence synthesis. PLoS One.

[30] Ellington M, Connelly J, Clayton P (2022). Use of Facebook, Instagram, and Twitter for recruiting healthy participants in nutrition-, physical activity-, or obesity-related studies: a systematic review. Am J Clin Nutr.

[31] Frampton GK, Shepherd J, Pickett K, Griffiths G, Wyatt JC (2020). Digital tools for the recruitment and retention of participants in randomised controlled trials: a systematic map. Trials.

[32] National Academies of Sciences, Engineering, and Medicine, Policy and Global Affairs, Committee on Women in Science, Engineering, and Medicine, Committee on Improving the Representation of Women and Underrepresented Minorities in Clinical Trials and Research (2022). Improving Representation in Clinical Trials and Research: Building Research Equity for Women and Underrepresented Groups.

